# Local relapse of nasopharyngeal cancer and Voxel-based analysis of FMISO uptake using PET with semiconductor detectors

**DOI:** 10.1186/s13014-017-0886-9

**Published:** 2017-09-06

**Authors:** Yukiko Nishikawa, Koichi Yasuda, Shozo Okamoto, Yoichi M. Ito, Rikiya Onimaru, Tohru Shiga, Kazuhiko Tsuchiya, Shiro Watanabe, Wataru Takeuchi, Yuji Kuge, Hao Peng, Nagara Tamaki, Hiroki Shirato

**Affiliations:** 1 Department of Radiation Medicine, Graduate School of Medicine, Hokkaido University, North-15 West-7, Sapporo, Japan; 2 Department of Nuclear Medicine, Graduate School of Medicine, Sapporo, Japan; 3 Department of Biostatistics, Graduate School of Medicine, Sapporo, Japan; 4 Global Station for Quantum Medical Science and Engineering, Global Institution for Collaborative Research and Education, Sapporo, Japan; 5 Central Institute of Isotope Science, Hokkaido University, Sapporo, Japan; 6 Research & Development Group, Hitachi, Ltd., Kokubunji, Tokyo Japan; 7 Stanford University, Stanford, CA USA

**Keywords:** [^18^F]fluoromisonidazole, Positron emission tomography, Hypoxia, Intensity-modulated radiotherapy, Nasopharyngeal carcinoma

## Abstract

**Background:**

Hypoxic cancer cells are thought to be radioresistant and could impact local recurrence after radiotherapy (RT). One of the major hypoxic imaging modalities is [^18^F]fluoromisonidazole positron emission tomography (FMISO-PET). High FMISO uptake before RT could indicate radioresistant sites and might be associated with future local recurrence. The predictive value of FMISO-PET for intra-tumoral recurrence regions was evaluated using high-resolution semiconductor detectors in patients with nasopharyngeal carcinoma after intensity-modulated radiotherapy (IMRT).

**Methods:**

Nine patients with local recurrence and 12 patients without local recurrence for more than 3 years were included in this study. These patients received homogeneous and standard doses of radiation to the primary tumor irrespective of FMISO uptake. The FMISO-PET image before RT was examined via a voxel-based analysis, which focused on the relationship between the degree of FMISO uptake and recurrence region.

**Results:**

In the pretreatment FMISO-PET images, the tumor-to-muscle ratio (TMR) of FMISO in the voxels of the tumor recurrence region was significantly higher than that of the non-recurrence region (*p* < 0.0001). In the recurrent patient group, a TMR value of 1.37 (95% CI: 1.36–1.39) corresponded to a recurrence rate of 30%, the odds ratio was 5.18 (4.87–5.51), and the area under the curve (AUC) of the receiver operating characteristic curve was 0.613. In all 21 patients, a TMR value of 2.42 (2.36–2.49) corresponded to an estimated recurrence rate of 30%, and the AUC was only 0.591.

**Conclusions:**

The uptake of FMISO in the recurrent region was significantly higher than that in the non-recurrent region. However, the predictive value of FMISO-PET before IMRT is not sufficient for up-front dose escalation for the intra-tumoral high-uptake region of FMISO. Because of the higher mean TMR of the recurrence region, a new hypoxic imaging method is needed to improve the sensitivity and specificity for hypoxia.

**Electronic supplementary material:**

The online version of this article doi: (10.1186/s13014-017-0886-9) contains supplementary material, which is available to authorized users.

## Background

Hypoxic tumor cells are known to be radioresistant [1]. Clinical studies have shown that tumors with intra-tumoral hypoxia have a poor prognosis [2, 3]. Several strategies have been investigated to overcome tumor hypoxia, such as radiotherapy (RT) with hyperbaric oxygen therapy and hypoxic cell radio-sensitizers, and reports have indicated the usefulness of these approaches [4, 5]. However, because of their adverse effects, the difficulty of patient selection, and several other limitations, these strategies have not seen wide clinical use [6].

Intensity-modulated radiotherapy (IMRT) with up-front dose escalation to hypoxic subvolumes is a potential method of overcoming tumor hypoxia [7]. The remarkable development of imaging and external beam radiotherapy has made dose escalation to small subvolumes in the primary tumor, or dose painting, technically possible [8]. Hypoxia imaging, such as [^18^F]fluoromisonidazole (FMISO)-positron emission tomography (PET), can detect tumor hypoxia non-invasively [9], and it can be registered with computed tomography (CT) and magnetic resonance imaging (MRI). However, whether local recurrences occur inside the intra-tumoral hypoxic region detected by the FMISO-PET imaging after homogeneous dose irradiation remains to be determined. Analyses using conventional FMISO-PET have been conducted to address this question; however, because of blurred, low-resolution images, definitive conclusions have not been reached [10].

Today, however, the resolution of PET has improved considerably. We have previously shown the excellent performance of PET with semiconductor detectors (semiconductor PET) [11], and compared with conventional PET, this method has shown improvements in spatial resolution (2.3 mm vs. 4.6 mm), energy resolution (4.1% vs. 14.0%), and scatter fraction (23% vs. 37.5%) [12]. This modality can detect the intra-tumoral inhomogeneity more precisely than the conventional scintillator PET for head and neck cancer [13]. A second difficulty is obtaining reproducible FMISO images of the hypoxic region in the same patient [14]. This problem has already been overcome by using high spatial resolution PET imaging to take the images 4 h after (rather than 2.5 h after) the FMISO injection [15].

In this study, we investigated whether locally recurrent nasopharyngeal carcinoma (NPC) would occur in the high-uptake region of FMISO-PET imaging after prescribing a homogeneous dose of irradiation to primary tumors independent of the FMISO uptake. The resulting data were analyzed, and we expected to determine the amount of FMISO uptake in primary tumors as a threshold for the focal dose escalation using up-front dose painting IMRT for patients with NPC.

## Methods

### Patients

**Fig. 1:**
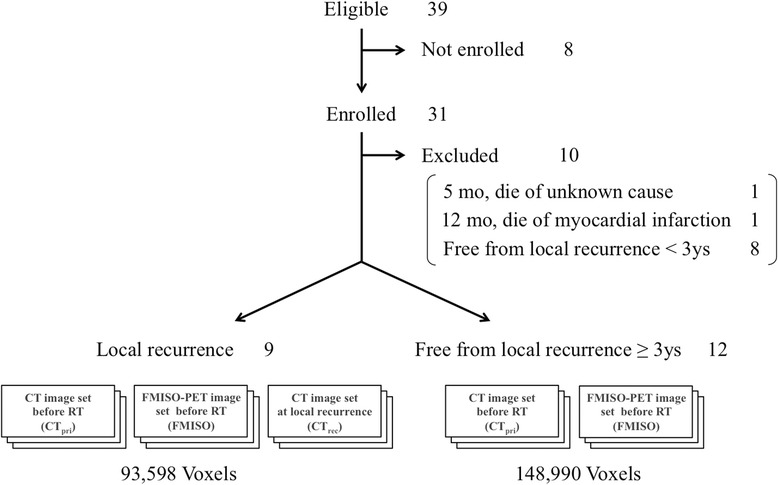
Number of patients and medical imaging modalities analyzed in this study. *Abbreviations: CT*, computed tomography; *RT*, radiation therapy; *CT*_*pri*_, CT before radiation therapy; *FMISO-PET*, ^18^F–fluoromisonidazole positron emission tomography; *CT*_*rec*_, CT at local recurrence

Thirty-nine patients were treated for NPC with definitive IMRT in our institution between April 2008 and December 2014 (Fig. 1). FMISO-PET was performed in 31 patients before starting RT. The other 8 patients did not receive FMISO-PET because they declined it or their schedules did not allow it. All 31 patients completed a course of RT and had been regularly followed-up at our outpatient clinic. Magnetic resonance imaging and/or CT was performed every 3 or 4 months and [^18^F]fluorodeoxyglucose (FDG)-PET was performed every 6 months at their follow-up examinations. Two patients who died of other causes (1 myocardial infarction at 12 months and 1 unknown cause at 5 months) and 8 patients who were followed-up on for less than 36 months by December 2015 without local recurrence were not included in the following analysis.

Thus, the remaining 21 patients were used for the analysis. The recurrent group consisted of 9 patients who experienced local recurrence with a median recurrence time of 11 months (range 5 to 46 months) from the start of RT. The non-recurrent group consisted of 12 patients without local recurrence with a median follow-up period of 46.5 months (range 37–61 months) (Table 1). The diagnosis of local recurrence was confirmed by a histological examination for 5 of 9 patients. The diagnosis was performed clinically with more than two image modalities for the other 4 patients because a tissue biopsy was considered impossible by otolaryngologists or neurosurgeons.

**Table 1:** Characteristics of 9 recurrent and 12 non-recurrent patients

Patient No.	Age (y)	Sex	Local Recur-rence	Time to Local Recurrence (m)	Follow-up Period (m)	TNM Classification (UICC, 7th edition)				WHO Histological Classification	Radiotherapy		Chemo-therapy	Number of Voxels	Number of Overlapped Voxels
						T	N	M	Stage		Dose (Gy)	Frac-tion			
1	57	M	+	5		3	2	0	III	NA	70	35	+	13,758	4410
2	45	F	+	7		2	2	0	III	III	70	35	+	10,954	1679
3	61	M	+	9		2	2	0	III	II or III	70	35	+	2235	906
4	48	M	+	9		1	1	0	II	III	70	35	+	1867	868
5	66	M	+	11		3	0	0	III	II or III	70	35	+	11,981	4116
6	61	M	+	16		4	0	0	IVA	I	70	35	+	12,388	2187
7	59	F	+	23		4	1	0	IVA	II	66	33	+	18,197	153
8	40	M	+	30		3	1	0	III	II or III	70	35	+	18,681	462
9	66	F	+	46		3	0	0	III	III	70	35	+	3537	1832
1	67	M	–		37	4	2	0	IVA	II or III	70	35	+	11,976	
2	61	M	–		40	2	2	0	III	NA	70	35	+	5610	
3	45	F	–		41	4	1	0	IVA	III	70	35	+	18,610	
4	44	M	–		43	3	2	0	III	III	70	35	+	8478	
5	53	M	–		44	3	2	0	III	II or III	70	35	+	30,802	
6	63	M	–		45	1	2	0	III	II or III	70	35	+	4116	
7	77	M	–		48	1	0	0	I	NA	70	35	–	3756	
8	50	F	–		50	2	2	0	III	I	70	35	+	39,955	
9	52	F	–		53	1	1	0	II	II or III	70	35	+	1220	
10	52	M	–		54	3	2	0	III	II or III	70	35	+	16,309	
11	77	M	–		60	2	2	0	III	II or III	70	35	–	5498	
12	53	M	–		61	2	1	0	II	II or III	70	35	+	2660	

*Abbreviation: NA* not available

### Outline of our new PET scanner

The diameter of the patient port is 350 mm, the transaxial field of view (FOV) is 310 mm, and the axial FOV is 246 mm. Eighteen detector units are radially arranged around the patient port. The detector size is 2 × 4 × 7.5 mm, and the dimensions of the detector unit are 100 × 400 × 350 mm. In the unit, the detector boards are arranged in parallel, and the detectors are mounted on both sides of each board. Each detector board has 96 detectors on each side (192 detectors in total) and signal processors. Signals are read by a 3-layer depth-of-interaction (DOI) system. Each unit has 22 boards and approximately 4000 detectors. The energy resolution of the scanner is 4.1% (full width at half maximum [FWHM]), which is superior to the energy resolution obtained with available scintillation detectors (i.e., 10–20%). The transverse and axial resolutions near the center are 2.3 and 5.1 mm, respectively, and correspond to National Electrical Manufacturers Association (NEMA) standards. The absolute sensitivity and the scatter fraction of the scanner evaluated with the NEMA NU 2–1994 phantom are 17.6 k counts per second (kcps)/ kilobecquerel (kBq)/mL and 23%, respectively. These estimates are for a lower-energy threshold of 450 kiloelectron volt (keV). The noise equivalent count rates (NEC2R) value is 41 kcps at 3.7 kBq/mL [11, 16].

Achieving a reduction in scatter noise required the energy window to be set at 490–530 keV (double FWHM of energy resolution). Images were reconstructed with a maximum a posteriori (MAP) reconstruction algorithm by applying the median root prior (MRP), which is known to be useful for edge preservation [16]. In addition, for the recovery of resolution, a measured point spread function was convolved with images and the system matrix in the MAP iterative process. The effects of reducing noise and recovering resolution can be controlled by choosing the degree of prior contribution. The proposed reconstruction method improved the image quality in terms of statistical noise and resolution. With the choice of a suitable degree of prior contribution, this method can improve the quality of images reconstructed from noisy or sparse data [16]. The voxel size of the reconstructed image was 1.21 × 1.21 × 2.8 mm for the PET examination in this study.

### FMISO-PET and other modalities

The protocol for conducting the FMISO-PET study was approved by the ethics review board at our institution in 2007. In this protocol, FMISO-PET was performed in NPC patients before definitive IMRT. Written informed consent was obtained from all patients before the FMISO-PET examination. Semiconductor PET was used for FMISO-PET in all patients. The patients were injected with 400 MBq of FMISO intravenously and imaged 4 h later. CT, MRI, and FDG-PET were performed before RT and at the follow-up examination.

### Radiotherapy and chemotherapy

All 21 patients were treated with IMRT. The clinical target volume 1 (CTV1) was defined as the volume that included the gross tumor volume (GTV) and the surrounding microscopic disease at risk. The planning target volume 1 (PTV1) was generated by adding a 3 mm margin around the CTV1. The cervical lymph node regions in the high-risk and low-risk areas were defined as the CTV2 and CTV3, respectively. The PTV2 and PTV3 were generated in the same manner as the PTV1. The prescribed doses to the PTVs and the dose constraints to organs at risk (OARs) are shown in Additional files 1 and 2: Tables S1 and S2. Seventy gray in 35 fractions was prescribed to the PTV1s except in 1 patient in the recurrent group who received 66 Gy in 33 fractions.

Chemotherapy was given to 19 of 21 patients. Two patients were treated by RT alone without any chemotherapy. Induction chemotherapy was administered to 6 patients, concurrent chemotherapy was administered to 19 patients, and adjuvant chemotherapy was administered to 9 patients. The induction chemotherapy drugs docetaxel (75 mg/m^2^, day 1), cisplatin (CDDP, 75 mg/m^2^, day 1), and 5-fluorouracil (5-FU, 750 mg/m^2^, day 1–5) were administered for 1–3 cycles for 5 patients and tegafur/gimeracil/oteracil (S-1, 100 mg/body, 2 weeks) was administered for 1 cycle for 1 patient. The concomitant chemotherapy drug CDDP (40 mg/m^2^, weekly) was administered for 1–6 cycles. The adjuvant chemotherapy drugs CDDP (80 mg/m^2^, day 1) and 5-FU (800 mg/m^2^, day 1–5; FP) were administered for 2–3 cycles for 7 patients; FP was administered for 2 cycles and S-1 was administered for 15 cycles for 1 patient; and S-1 was administered for 4 or 11 cycles for 2 patients. Details of the chemotherapy are shown in Additional file 3: Table S3.

### Tumor-to-muscle ratio (TMR) of FMISO

The FMISO-PET image set was imported into the image-analysis software VOX-BASE (J-MAC System, Sapporo, Japan). The voxel size was calculated as 1 × 1 × 2 mm from the slice thickness and field of view. We assumed that each voxel is independent and does not influence the other voxels. The value of a voxel represented the standardized uptake volume.

Regions of interest (ROIs) with a radius of 1 cm were placed in four positions on the posterior cervical muscles on FMISO-PET images of each patient, with laterally displayed or fused CT images used as the reference for localization. The maximum standardized uptake value (SUV_max_) in these 4 ROIs was measured, and their mean was calculated and used as the SUV_max_ for the posterior cervical muscles. Then, the SUV in each voxel was divided by the SUV_max_ for the muscles, and the resulting value was defined as the TMR.

### Voxel-based image analysis

All 9 patients in the recurrent group were examined by planning CT, MRI, [^18^F]FDG-PET and FMISO-PET within a median (range) of 24.5 (1–50) days before the start of RT. Planning CT, [^18^F]FDG-PET and FMISO-PET were performed using plastic immobilization devices. MRI was performed without immobilization devices. At the local recurrence region, MRI and FDG-PET were performed on all patients and CT was performed on 8 of 9 patients. All 12 patients in the non-recurrent group were examined by planning CT, MRI, [^18^F]FDG-PET and FMISO-PET within a median of 16.5 (2–87) days before the start of RT.

**Fig. 2:**
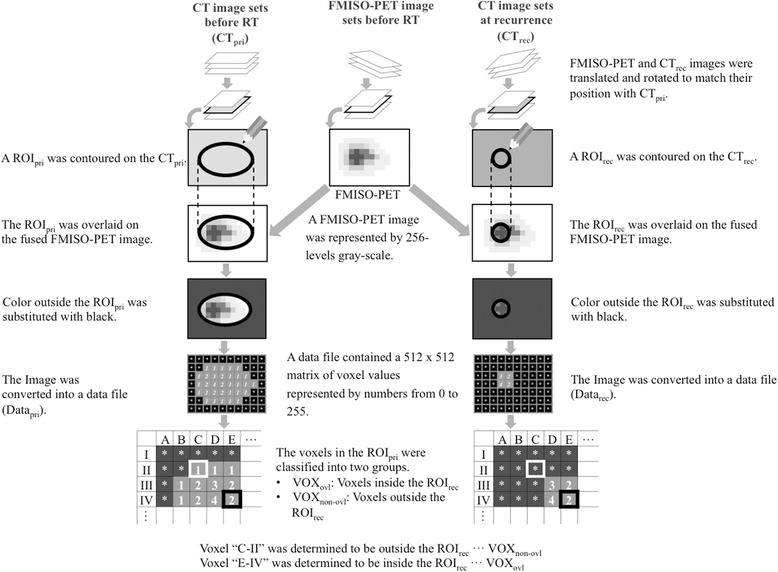
Voxel-based image analysis procedures. *Abbreviations: CT*, computed tomography; *RT*, radiation therapy; *CT*_*pri*_, CT before radiation therapy; *FMISO-PET*, ^18^F–fluoromisonidazole positron emission tomography; *CT*_*rec*_, CT at local recurrence; *ROI*_*pri*_, region of interest designated primary tumor; *ROI*_*rec*_, region of interest designated recurrent tumor; *Data*_*pri*_, the data files derived from the ROI_pri_; *Data*_*rec*_, the data files derived from the ROI_rec_; *VOX*_*ovl*_, overlap voxels; *VOX*_*non-ovl*_, non-overlap voxels

The procedures below were performed on both groups, and the process is shown in Fig. 2.

1. All image sets were imported into an in-house image fusion software package. FMISO-PET image sets were translated and rotated to match their position with CT image sets before RT (CT_pri_) according to the rigid registration algorithm referencing normal anatomical structures and not the tumorous structure. In the recurrent group, CT image sets at local recurrence (CT_rec_) were also translated and rotated to match their position with CT_pri_ using the normal anatomical structures, mainly bone landmarks, for the registration. We checked the tumor position and the quality of the rigid registration between the CT_pri_ and CT_rec_. The representative plane images before RT and at recurrence for all recurrent patients are shown in Additional file 4: Figure S1. We judged that the quality of the rigid registration using bone landmarks was sufficient for all patients.
2. The registered image sets were imported into the open source software ImageJ [17].
3. Primary tumors before RT were contoured on CT_pri_ and referenced to MRI and FDG-PET images by a board-certified radiation oncologist. The ROI was designated the ROI_pri_, and it was consistent with the GTV. The median number of voxels in the ROI_pri_ in each patient was 10,954 (range from 1220 to 39,955) in all 21 patients.
4. In the recurrent group, recurrent tumors were contoured on another CT_pri_ and referenced to MRI, FDG-PET and CT_rec_ images by the same board-certified radiation oncologist. The ROI was designated the ROI_rec_. The median number of voxels in the ROI_rec_ in each recurrent patient was 1679 (range from 153 to 4410).
5. The ROI_pri_ and ROI_rec_ were separately overlaid on the registered FMISO-PET image sets. The FMISO-PET images were represented by a 256-level grayscale.
6. Referring to the ROI_pri_ or ROI_rec_, we changed any colors outside the ROI_pri_ or ROI_rec_ on the FMISO-PET images to black because this study was focused on the question of whether FMISO uptake inside primary tumors was related to the risk of local recurrence. Whether the FMISO uptake outside primary tumors is related to the risk of local recurrence is beyond the scope of this study.
7. The FMISO-PET images were converted into data files that consisted of the FMISO uptake values in each voxel. The data files derived from the ROI_pri_ and ROI_rec_ were named Data_pri_ and Data_rec_, respectively. A data file contained a 512 × 512 matrix of voxel values represented by the numbers 0 to 255. The value of the black area around each ROI was 0, which indicated that these voxels were outside the primary tumors and should be excluded from the analysis.
8. The Data_pri_ and Data_rec_ values were compared in the same spatial coordination space. For each voxel, whether the voxels occurred inside the primary or recurrent tumor was determined. The voxels in ROI_pri_ were classified into two groups: overlap voxels (VOX_ovl_) and non-overlap voxels (VOX_non-ovl_). VOX_ovl_ consisted of the voxels inside both the ROI_rec_ and ROI_pri_, and VOX_non-ovl_ consisted of the voxels outside the ROI_rec_ but inside the ROI_pri_.
9. In the non-recurrent group, ROI_rec_ was not generated and only Data_pri_ values were available. As a result, all voxels in the ROI_pri_ were classified as VOX_non-ovl_.

### Mean and minimum doses in primary tumors

The mean and minimum doses (D_mean_, D_min_) in the primary tumors, which were equal to the GTVs, were measured using three-dimensional radiation therapy planning (RTP) software (Pinnacle^3^ version 80 m; Philips Radiation Oncology Systems, Fitchburg, WI). The differences in D_mean_ and D_min_ between the recurrent and non-recurrent groups were analyzed***.***

### Statistical analysis

The Mann-Whitney U test was used to determine statistical significance. A logistic regression analysis was performed to evaluate the predictive abilities of FMISO-PET when local recurrence did or did not occur in voxels inside the primary tumors. The receiver operating characteristic (ROC) curve for the estimated logistic regression model was generated to assess the model’s ability to identify voxels that experienced local recurrence. A *p*-value less than 0.05 was considered statistically significant. Analyses were conducted using the software packages JMP®12 (SAS Institute Inc., Cary, NC, USA) and R version 3.3.1 [18].

## Results

**Fig. 3:**
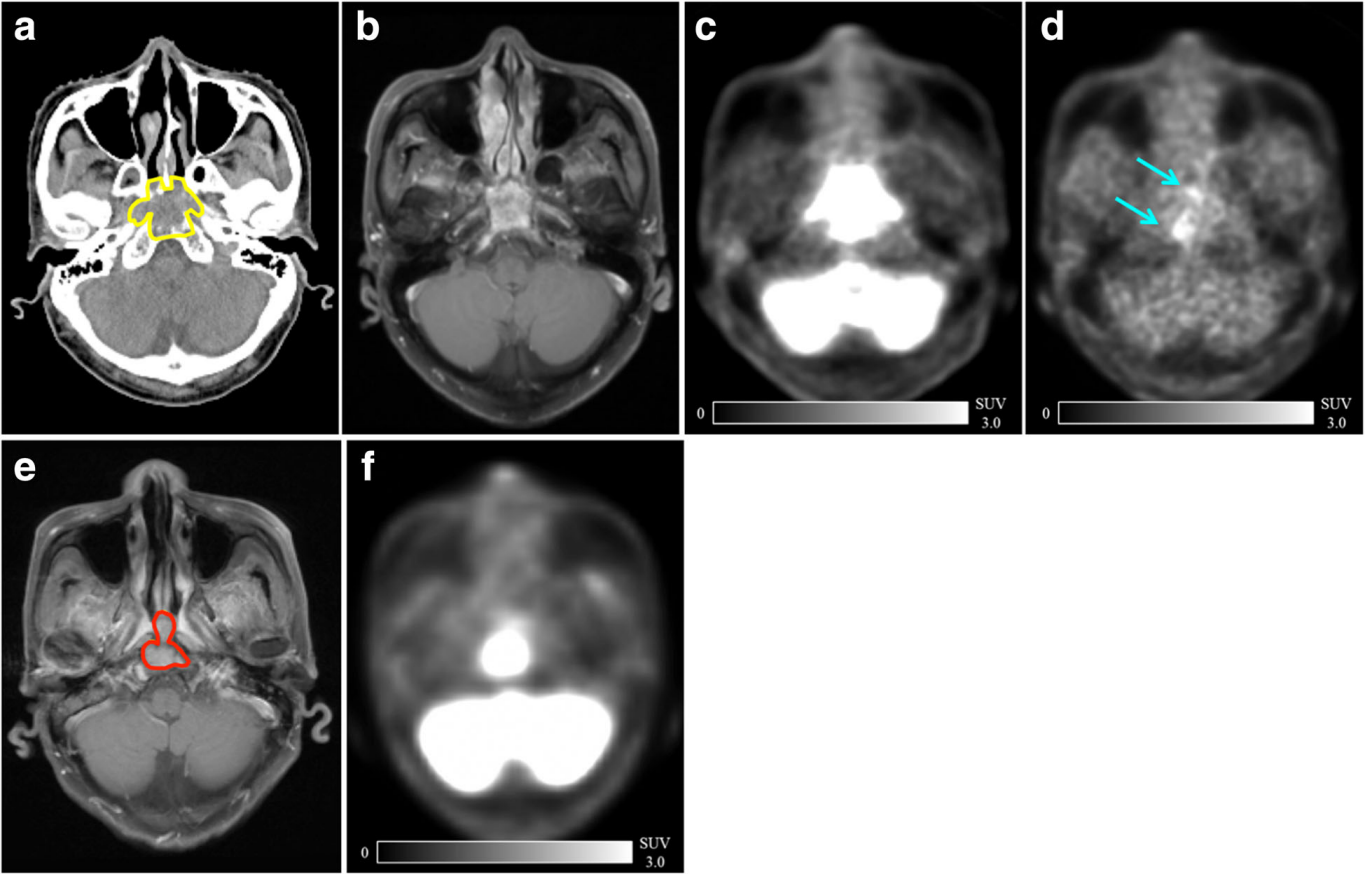
Representative cases. CT (**a**), contrast-enhanced MRI (**b**), FDG-PET (**c**) and FMISO-PET (**d**) were performed before radiation therapy. The CT showed nasopharyngeal mucosal disease and invasion to the clivus (yellow line). The high-uptake area of FMISO was distinguished at the central part of mucosal region and slightly to the right side of the clivus (cyan arrow). Five months after chemoradiation, this patient experienced local relapse. Contrast-enhanced MRI (**e**) and FDG-PET (**f**) showed recurrent tumor (red line). The relapse site was central to the nasopharyngeal mucosa and clivus and of a similar position with the pretreatment FMISO uptake area. *Abbreviations: CT*, computed tomography; *FDG-PET*, ^18^F- fluorodeoxyglucose positron emission tomography; *FMISO-PET*, ^18^F–fluoromisonidazole positron emission tomography

The representative case is illustrated in Fig. 3. The locational relationship between the pretreatment FMISO-PET and relapse site was estimated in this patient.

The maximum TMR (TMR_max_) of FMISO uptake in the primary tumors (ROI_pri_) was compared between the recurrent and non-recurrent groups. The mean TMR_max_ was 1.86 (0.169 standard error [SE]) in the recurrent group and 1.94 (0.181 SE) in the non-recurrent group, and these values were not significantly different (*p* = 0.776).

**Fig. 4:**
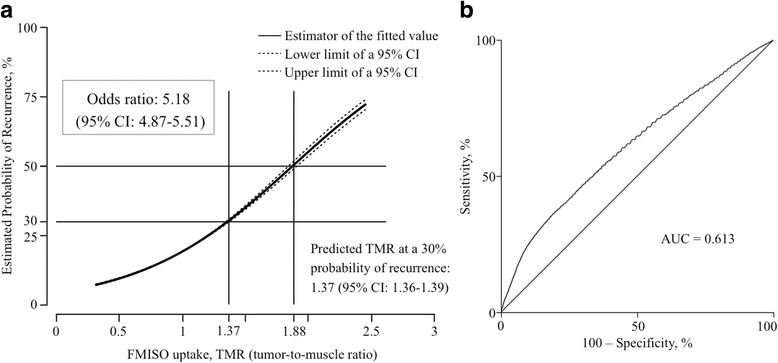
Logistic regression analysis of the relationship between the TMR in each voxel and the probability of local recurrence in that voxel (**a**), and receiver operating characteristic curve (**b**) for the recurrent group. *Abbreviations: CI* confidence interval, *TMR* tumor-to-muscle ratio, *FMISO* ^18^F–fluoromisonidazole, *AUC* area under the curve

In the recurrent group, 93,598 voxels were observed in total, with 16,613 voxels in the VOX_ovl_ and 76,985 voxels in the VOX_non-ovl_. The mean TMR was 1.01 (0.00234 SE) in the VOX_ovl_ and 0.898 (0.00083 SE) in the VOX_non-ovl_. The TMR in the VOX_ovl_ was significantly higher than that in the VOX_non-ovl_ (*p* < 0.0001). In the recurrent group, the logistic curve showed that the probability of local recurrence increased according to the increase in the TMR of the voxel (Fig. 4a). The estimated TMR value at a 50% probability of local recurrence was 1.88 (95% CI: 1.85–1.92). The estimated TMR value at 30% probability of local recurrence was 1.37 (95% CI: 1.36–1.39). The odds ratio was 5.18 (95% CI: 4.87–5.51), and the area under the curve (AUC) of the ROC curve was 0.613 (Fig. 4b). Among the total 93,598 voxels, the number of voxels with TMRs of greater than 1.88 and 1.37 was 484 (0.52%) and 5880 (6.28%), respectively.

**Fig. 5:**
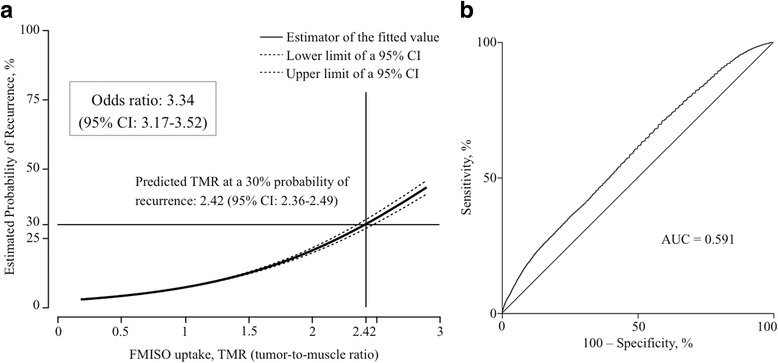
Logistic regression analysis of the relationship between TMR and probability of local recurrence (**a**) and receiver operating characteristic curve (**b**) for all patients. *Abbreviations: CI*, confidence interval; *TMR*, tumor-to-muscle ratio; *FMISO*, ^18^F–fluoromisonidazole; *AUC*, area under the curve

Next, we re-performed the above analysis and included the 12 patients in the non-recurrent group. We observed 148,990 voxels in the VOX_non-ovl_ of the non-recurrent group, and these voxels were combined with the 76,985 voxels in the VOX_non-ovl_ of the recurrent group. The combined 225,975 voxels were designated non-overlap voxels in the whole series (_whl_VOX_non-ovl_). The mean TMR was 1.01 (0.00234 SE) in the VOX_ovl_ and 0.916 (0.00055 SE) in the _whl_VOX_non-vol_ when we combined the 9 recurrent patients and 12 non-recurrent patients. The TMR of the VOX_ovl_ was significantly higher than that of the _whl_VOX_non-vol_ (p < 0.0001). When we adapted the logistic curve for the whole series, the probability of local recurrence was 30% at a TMR value of 2.42 (95% CI: 2.36–2.49). The logistic curve for the whole series showed that the probability of local recurrence increased according to the increase in TMR as shown in Fig. 5a. The odds ratio was 3.34 (3.17–3.52), and the AUC of the ROC curve was 0.591 (Fig. 5b).

The means of the D_mean_ values in the primary tumors were 73.3 Gy (2.5 SE) and 74.3 Gy (0.9 SE) in the recurrent and non-recurrent groups, respectively. There was no significant difference between the two groups (*p* = 0.522). The means of the D_min_ values in the primary tumors were 64.3 Gy (3.7 SE) and 66.6 Gy (2.4 SE) in the recurrent and non-recurrent groups, respectively. Significant differences were not observed between the two groups (*p* = 0.356).

## Discussion

FMISO-PET has been shown to be useful for distinguishing tumor hypoxia from normal tissue in NPC patients [19]; however, previous studies have reported conflicting results as to whether the maximum FMISO value can predict recurrence [20, 21]. Our results suggested that the maximum FMISO uptake value, TMR_max_, is not useful for predicting local recurrence. We found that the TMR in the recurrent region, VOX_ovl_, was significantly higher than that in the non-recurrent region, _whl_VOX_non-vol_, not only when the analysis was limited to the patients in the recurrent group but also when the patients in the non-recurrent group were included (*p* < 0.0001 for both groups).

Our results suggested that the risk of local recurrence in a voxel increased according to the amount of FMISO uptake in the patients who experienced local recurrence. In the logistic regression analysis for these patients, when the TMR values in a voxel were 1.37 and 1.88, the risk of local recurrence in the voxel was 30 and 50%, respectively. Although the AUC of the ROC curve of 0.613 was not particularly impressive, a higher uptake of FMISO in a voxel was suggested to confer a higher risk of local recurrence in the same voxel and presented an odds ratio of 5.18.

In the logistic regression analysis for all 21 patients, the risk of local recurrence was 30% when the TMR in a voxel was 2.42. However, because the AUC of the ROC curve was as small as 0.591, we cannot reasonably conclude that the higher uptake of FMISO in a voxel indicates a higher risk of local recurrence at the same voxel with an odds ratio of 3.34. Thus, the present study suggests that the predictive value of FMISO-PET before RT is not sufficient for up-front dose escalation IMRT for the intra-tumoral high-uptake region of FMISO, even when using semiconductor PET.

However, it is important to note that a significant relationship was observed between the higher uptake and local recurrence in patients who actually experienced local recurrence. This finding implies a new imaging method with improved specificity for hypoxia should be developed. Such a method would need to have a higher AUC of the ROC curve than FMISO-PET. A hypoxic tracer that presents superior results relative to FMISO would be useful for determining the threshold for up-front dose escalation in IMRT at its high-uptake volume in patients with NPC. The present study has provided a platform for translational research for candidates for such a new hypoxic imaging modality.

In general, the signal-to-background ratio in FMISO-PET is not large. The general cut-off values of 1.2 to 1.4 were determined based on the uptake of FMISO in normal tissue. Rajendran JG et al. reported that >99% of normal tissues did not present uptake ≥1.2 in their animal and human studies; therefore, they set the cut-off value to 1.2 [22]. The cut-off value of 1.2–1.4 is reasonable for determining whether the specific region has a statistically higher uptake than normal distribution, although its clinical meaning has not been addressed. The TMR for a 30% probability of local recurrence was 1.37 in the recurrent group, which is in the range of the general cut-off value; therefore, general cut-off values can be used as a predictive guide for careful follow-up examinations of these patients. However, the TMR for a 30% probability of local recurrence was 2.42 for all studied patients; thus, using general cut-off values of 1.2–1.4 are not feasible for determining the region for dose escalation.

One of the essential limitations of this study is that we assumed in the discussion that the FMISO uptake in each voxel was not influenced by any factors other than hypoxia. These hypotheses should be carefully evaluated in the future. Another limitation concerns the registration of the images. Normal anatomical structures do not present large changes before and after the treatment, and tumors that invade the skull base also do not change position. However, tumors in the submucosal region can change shape and size; therefore, the hypoxic region at treatment may not be in the exact position upon recurrence with our registration method. As shown in Additional file 4: Figure S1, we judged the quality of rigid registration using bone landmark was sufficient for all patients in our series. However, it was also a fact that some mucosal changes were observed. The voxel analysis using non-rigid registration will be an important next step to improve the accuracy of this analysis. Our results only imply that our results were not able to support the use of FMISO-PET imaging for dose escalation to the high-uptake region, and biological interpretations related to the shrinkage of the tumor and the change of hypoxic region are beyond the scope of our study. Thus, additional studies are required to address this problem of the non-rigid registration method and tumor biology in the future [23]. The limited availability of semiconductor PET is another limitation for FMISO-PET. The chemotherapy of the 21 patients was highly variable and could represent a source of confronting bias, which is another limitation of this study.

Contrast and specificity may be improved by reducing background activity using new hypoxic tracers, such as [18F]-HX4 and [18F]DiFA [24, 25]. This study suggested that greater reductions in the background activity other than FMISO can improve the performance of hypoxia imaging for predicting local recurrence. These new tracers are expected to provide more information about hypoxia and be more useful for defining regions that will require greater doses to reduce local recurrence.

## Conclusions

The uptake of FMISO in the recurrent region was significantly higher than that in the non-recurrent region (*p* < 0.0001). However, the present study also suggested that the predictive value of FMISO-PET before RT was not sufficient for an up-front dose escalation IMRT to the intra-tumoral high-uptake region of FMISO, even when we used semiconductor PET. Therefore, a new hypoxic imaging method should be developed with better specificity for hypoxia and a higher AUC of the ROC curve compared with FMISO-PET.

## Additional files

Additional file 1: Table S1. The prescribed doses to the PTVs. *Abbreviations: PTV* planning target volume, *D*_*XX%*_ the maximum dose covering the target volume of XX%, *V*_*XXGy*_ the percent of the target volume receiving XXGy. (DOCX 40 kb)

Additional file 2: Table S2. The dose constraints to organs at risk (OARs). *Abbreviations: OAR* organ at risk, *PRV* planning organ at risk volume, *D*_*XX%*_ the maximum dose covering the target volume of XX%, *D*_*1cc*_ the maximum dose covering the target volume of 1 cm^3^, *V*_*XXGy*_ the percent of the target volume receiving XXGy, *D*_*max*_ maximum dose, *D*_*mean*_ mean dose, *D*_*median*_ median dose. (DOCX 47 kb)

Additional file 3: Table S3. Chemotherapy used in each patient. *Abbreviations: TPF* Docetaxel, Cisplatin and Fluorouracil, *S-1* Tegafur/Gimeracil/Oteracil, *CDDP* Cisplatin, *FP* Fluorouracil and Cisplatin. (DOCX 57 kb)

Additional file 4: Figure S1. The representative plane images before RT and at recurrence of all recurrent patients. Yellow line indicated primary tumor before RT, and red line indicated recurrence tumor. *Abbreviations: RT* radiation therapy. (PNG 1004 kb)
