# Supplementary material for: Local relapse of nasopharyngeal cancer and Voxel-based analysis of FMISO uptake using PET with semiconductor detectors
Source: Radiat Oncol. 2017 Sep 6;12:148. doi: 10.1186/s13014-017-0886-9 (PMC5586018; doi:10.1186/s13014-017-0886-9)
Supplement: Supplementary file 1 — The prescribed doses to the PTVs. Abbreviations: PTV planning target volume, D XX% the maximum dose covering the target volume of XX%, V XXGy the percent of the target volume receiving XXGy. (DOCX 40 kb) [file 13014_2017_886_MOESM1_ESM.docx]

Table S1 The prescribed doses to the PTVs

| **Target Volume** | **Dose** | **Criteria** | | | |
| --- | --- | --- | --- | --- | --- |
| PTV1 | 70 Gy | D_98%_ | > | 65.1 | Gy |
|  |  | D_95%_ | = | 70 | Gy |
|  |  | D_50%_ | < | 73.5 | Gy |
|  |  | D_10%_ | < | 77 | Gy |
|  |  | D_2%_ | < | 84 | Gy |
| PTV1  (for patient No.7) | 66 Gy | D_95%_ | = | 66 | Gy |
|  |  | V_72.6Gy_ | < | 20 | % |
|  |  | V_75.9Gy_ | < | 5 | % |
|  |  | V_61.4Gy_ | ≥ | 99 | % |
| PTV2 | 63 Gy | D_95%_ | ≥ | 63 | Gy |
| PTV3 | 56 Gy | D_95%_ | ≥ | 56 | Gy |
