# Supplementary material for: Local relapse of nasopharyngeal cancer and Voxel-based analysis of FMISO uptake using PET with semiconductor detectors
Source: Radiat Oncol. 2017 Sep 6;12:148. doi: 10.1186/s13014-017-0886-9 (PMC5586018; doi:10.1186/s13014-017-0886-9)
Supplement: Supplementary file 2 — The dose constraints to organs at risk (OARs). Abbreviations: OAR organ at risk, PRV planning organ at risk volume, D XX% the maximum dose covering the target volume of XX%, D 1cc the maximum dose covering the target volume of 1 cm3, V XXGy the percent of the target volume receiving XXGy, D max maximum dose, D mean mean dose, D median median dose. (DOCX 47 kb) [file 13014_2017_886_MOESM2_ESM.docx]

Table S2 The dose constraints to organs at risk (OARs)

| **Organ** | **Dose Constraint** |
| --- | --- |
| Spinal Cord_PRV | D_max_ < 50 Gy |
|  | D_1cc_ < 46 Gy |
| Spinal Cord | D_1cc_ < 46 Gy |
| Brain_PRV | D_max_ < 70 Gy |
| Brain - 5 mm | D_max_ < 60 Gy |
| Brain Stem_PRV | D_max_ < 54 Gy |
| Brain Stem | D_max_ < 54 Gy |
| Optic Nerve_PRV | D_max_ < 50 Gy |
| Eye | D_max_ < 40 Gy |
| Lens | D_mean_ < 6 Gy |
| Parotid | D_median_ < 20 Gy |
|  | D_mean_ < 26 Gy |
| Constrictor Muscles | D_mean_ < 54 Gy |
| Larynx | D_mean_ < 45 Gy |
| Inner Ear_PRV | D_mean_ < 45 Gy |
| Mandible | V_65Gy_ (as low as possible) |
|  | V_70 Gy_ (as low as possible) |
|  | V_75Gy_ < 1cc |
| Oral | D_mean_ < 45 Gy |
|  | V_60Gy_ (as low as possible) |
| Esophagus | V_45Gy_ (as low as possible) |
| Lung | V_20Gy_ (as low as possible) |
