# Supplementary material for: Local relapse of nasopharyngeal cancer and Voxel-based analysis of FMISO uptake using PET with semiconductor detectors
Source: Radiat Oncol. 2017 Sep 6;12:148. doi: 10.1186/s13014-017-0886-9 (PMC5586018; doi:10.1186/s13014-017-0886-9)
Supplement: Supplementary file 3 — Chemotherapy used in each patient. Abbreviations: TPF Docetaxel, Cisplatin and Fluorouracil, S-1 Tegafur/Gimeracil/Oteracil, CDDP Cisplatin, FP Fluorouracil and Cisplatin. (DOCX 57 kb) [file 13014_2017_886_MOESM3_ESM.docx]

Table S3 Chemotherapy used in each patient

| **Patient No.** | **Local Recurrence** | **Induction (cycle)** | **Concurrent (cycle)** | **Adjuvant (cycle)** |
| --- | --- | --- | --- | --- |
| 1 | + | TPF (1) | CDDP (6) | S-1 (4) |
| 2 | + | S-1 (1) | CDDP (4) | - |
| 3 | + | - | CDDP (4) | FP (2) |
| 4 | + | - | CDDP (5) | FP (2) |
| 5 | + | TPF (3) | CDDP (5) | - |
| 6 | + | - | CDDP (1) | S-1 (11) |
| 7 | + | TPF (2) | CDDP (1) | - |
| 8 | + | - | CDDP (3) | FP (2) |
| 9 | + | - | CDDP (4) | - |
| 1 | - | - | CDDP (2) | FP (3) |
| 2 | - | - | CDDP (4) | - |
| 3 | - | - | CDDP (3) | FP (3) |
| 4 | - | - | CDDP (2) | FP (3) |
| 5 | - | - | CDDP (2) | FP (3) + S-1 (15) |
| 6 | - | - | CDDP (3) | FP (3) |
| 7 | - | - | - | - |
| 8 | - | TPF (1) | CDDP (4) | - |
| 9 | - | TPF (2) | CDDP (2) | - |
| 10 | - | - | CDDP (6) | - |
| 11 | - | - | - | - |
| 12 | - | - | CDDP (5) | - |

Abbreviation: TPF, Docetaxel + Cisplatin + Fluorouracil; S-1, Tegafur/Gimeracil/Oteracil; CDDP, Cisplatin; FP, Fluorouracil + Cisplatin.
