# Supplementary figures and images for: Local relapse of nasopharyngeal cancer and Voxel-based analysis of FMISO uptake using PET with semiconductor detectors
Source: Radiat Oncol. 2017 Sep 6;12:148. doi: 10.1186/s13014-017-0886-9 (PMC5586018; doi:10.1186/s13014-017-0886-9)

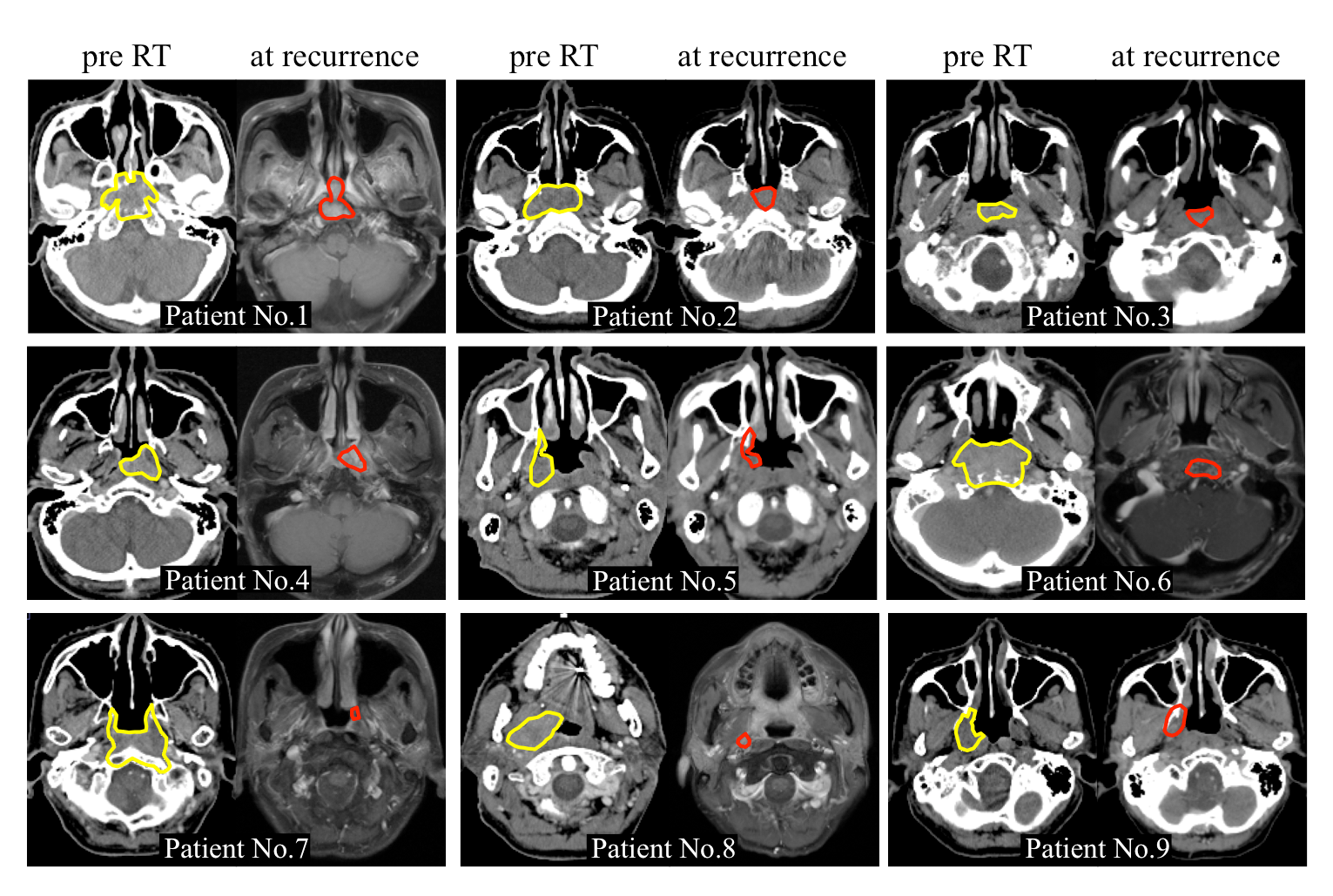

Supplement: Supplementary file 4 — The representative plane images before RT and at recurrence of all recurrent patients. Yellow line indicated primary tumor before RT, and red line indicated recurrence tumor. Abbreviations: RT radiation therapy. (PNG 1004 kb) [file 13014_2017_886_MOESM4_ESM.png]
